# New tissue priors for improved automated classification of subcortical brain structures on MRI^☆^

**DOI:** 10.1016/j.neuroimage.2016.01.062

**Published:** 2016-04-15

**Authors:** S. Lorio, S. Fresard, S. Adaszewski, F. Kherif, R. Chowdhury, R.S. Frackowiak, J. Ashburner, G. Helms, N. Weiskopf, A. Lutti, B. Draganski

**Affiliations:** a LREN, Department of Clinical Neurosciences, CHUV, University of Lausanne, Lausanne, Switzerland; b Medical Radiation Physics, Lund University Hospital, Lund, Sweden; c Wellcome Trust Centre for Neuroimaging, UCL Institute of Neurology, London, UK; d Max Planck Institute for Human Cognitive and Brain Sciences, Leipzig, Germany

**Keywords:** Relaxometry, Magnetization transfer saturation, Effective transverse relaxation, Basal ganglia, Tissue probability maps, Voxel-based morphometry, Voxel-based quantification

## Abstract

Despite the constant improvement of algorithms for automated brain tissue classification, the accurate delineation of subcortical structures using magnetic resonance images (MRI) data remains challenging. The main difficulties arise from the low gray-white matter contrast of iron rich areas in T1-weighted (T1w) MRI data and from the lack of adequate priors for basal ganglia and thalamus. The most recent attempts to obtain such priors were based on cohorts with limited size that included subjects in a narrow age range, failing to account for age-related gray-white matter contrast changes. Aiming to improve the anatomical plausibility of automated brain tissue classification from T1w data, we have created new tissue probability maps for subcortical gray matter regions. Supported by atlas-derived spatial information, raters manually labeled subcortical structures in a cohort of healthy subjects using magnetization transfer saturation and R2* MRI maps, which feature optimal gray-white matter contrast in these areas. After assessment of inter-rater variability, the new tissue priors were tested on T1w data within the framework of voxel-based morphometry. The automated detection of gray matter in subcortical areas with our new probability maps was more anatomically plausible compared to the one derived with currently available priors. We provide evidence that the improved delineation compensates age-related bias in the segmentation of iron rich subcortical regions. The new tissue priors, allowing robust detection of basal ganglia and thalamus, have the potential to enhance the sensitivity of voxel-based morphometry in both healthy and diseased brains.

## Highlights

• We create new tissue probability maps of subcortical structures based on magnetization transfer saturation and R2* MRI data.
• We obtain anatomically plausible delineation of subcortical structures from T1w data with the new tissue probability maps.
• Automated tissue classification with the new tissue probability maps is more robust against the age impact on MR contrast.

## Introduction

Computer-based assessment of brain anatomy with magnetic resonance imaging (MRI) has become a powerful method to investigate *in vivo* the healthy and diseased brain. Aiming to provide reliable estimates of local gray matter (GM) volume across the whole brain, a substantial amount of work has been devoted to the improvement of the accuracy of algorithms for automated tissue classification and spatial registration (Ashburner and Friston, 2000, Ashburner and Friston, 2005, Klein et al., 2010). Despite major methodological advances, the robust and accurate delineation of the deep brain nuclei – thalamus, caudate, putamen, pallidum, subthalamic nucleus, substantia nigra, and red nucleus – remains challenging (Lim et al., 2013, Streitbürger et al., 2014, Callaert et al., 2014). The basal ganglia play a crucial role in goal-directed behavior and movement control, which explains their involvement in many neurological and neuropsychiatric disorders such as Parkinson's and Huntington's disease, dystonia, tremor, Tourette's syndrome, and schizophrenia (Utter and Basso, 2008). The reliable anatomical assessment of these regions is important not only to accurately monitor disease-related changes but also to facilitate accurate target identification for functional neurosurgery in basal ganglia disorders. There is therefore a clear need to improve the automated detection of basal ganglia structures (Ahsan et al., 2007).

Automated tissue classification relies on the distributions of image intensities and gray-white matter contrast in MRI images (Ashburner et al., 2003), which are determined by the local values of the MRI parameters and the microstructural composition of brain tissue (Fukunaga et al., 2010, Streitbürger et al., 2014, Lutti et al., 2014). In particular, the inaccurate classification of subcortical structures from T1-weighted (T1w) images—the most widely used data in computational anatomy, arises from the high concentration of iron in these regions (Hallgren and Sourander, 1958, Haacke et al., 2005, Lorio et al., 2014). Importantly, this effect is further modulated by age-related tissue property changes (Lorio et al., 2014).

In addition to its dependence on image intensity and gray-white matter contrast, the automated tissue classification relies on prior spatial information based either on stereotaxic atlases (Fischl et al., 2002, Pohl et al., 2006, Khan et al., 2008) or on probabilistic maps of tissue class distributions derived from MRI data (Ashburner and Friston, 2005). The currently used tissue probability maps are based on T1w data (Mazziotta et al., 2001) with the major drawback of a regional contrast differences driven by microstructural tissue properties (Lorio et al., 2014). More recent attempts to improve the priors for robust classification of subcortical structures have benefited from new MRI protocols that highlight the impact of tissue properties on gray-white matter contrast. These recent achievements are limited by the relatively low number of used data samples, which hampers the accurate detection of inter-individual variations in brain anatomy and their modulation by age (Ahsan et al., 2007, Prodoehl et al., 2008, Lim et al., 2013, Keuken et al., 2014). Common to the previous studies on the topic is that there was no attempt to statistically assess the impact of new anatomically plausible tissue probability maps on the automated tissue classification within computational anatomy frameworks.

The purpose of this study is to build new tissue probability maps (TPMs) for the automated tissue classification of thalamus, caudate, putamen, globus pallidus, substantia nigra, subthalamic nucleus, red nucleus, and cerebellar dentate. The new TPMs were derived from the manual labeling of subcortical structures on magnetization transfer saturation (MT) and R2* (= 1/T2*) maps, which provide optimal contrast in these areas (Helms et al., 2009). The obtained TPMs were then included as a new tissue prior in the Bayesian framework for tissue classification of the well-established SPM software (Ashburner and Friston, 2005). To test the anatomical accuracy of the tissue classification performed with the new TPMs, we carried out a cross-validation between the manual labeling results and the gray matter volume maps obtained from the automated tissue classification based on MT images. Finally, the new TPMs were applied on an independent data set of T1w images. Our hypothesis was that the new tissue probability maps would enable the accurate delineation of subcortical structures and would prove particularly robust against the effects of age-related microstructural tissue changes on T1w data.

## Methods

### Data acquisition

We used quantitative MRI (qMRI) data for the manual labeling of subcortical structures. The qMRI images were originally acquired for previous studies (Chowdhury et al., 2013, Lorio et al., 2014). The data set comprised 96 healthy adults (40 male, age range 27–74 years, mean 55 ± 15; 56 female, age range 21–88 years, mean 57 ± 19) scanned on a 3 T whole-body MRI system (Magnetom TIM Trio, Siemens Medical Systems, Germany), using a standard 32-channel radio-frequency receive head coil and body coil for transmission. On visual inspection, study participants showed neither macroscopic brain abnormalities, i.e., major atrophy, nor signs of overt vascular pathology, i.e., micro-bleeds and white matter lesions. Elderly subjects with white matter lesions of Grade 2 or more by the Scheltens' rating scale (Scheltens et al., 1993, Wardlaw et al., 2013) were excluded from the study. We obtained quantitative measures of brain atrophy by calculating the brain volume fraction (Rudick et al., 1999) from MT images.

The quantitative MRI acquisitions consisted of three multi-echo 3D fast low angle shot (FLASH) acquired with predominant proton density, PD-, T1-, and MT-weighting (PD-weighted: TR/α = 23.7 ms/6°; T1-weighted: TR/α = 18.7 ms/20°; MT-weighted: TR/α = 23.7 ms/6°) with 1 mm^3^ isotropic resolution (Helms et al., 2008a, Weiskopf et al., 2013). The MT-weighting was achieved by applying an off-resonance Gaussian-shaped pulse (4 ms duration, 220 nominal flip angle, 2 kHz frequency offset from water resonance) prior to the excitation. Multiple gradient echoes were acquired for each FLASH acquisition with alternating readout polarity: 6 equidistant echo time (TE) were used for the T1- and MT-weighted sets (TE between 2.34 ms and 14.7 ms) and 8 equidistant TE were used for PD-weighted sets (TE between 2.34 ms and 19.7 ms). The image resolution was 1 mm isotropic. To shorten the acquisition time, parallel imaging (acceleration factor 2, GRAPPA), and partial Fourier acquisition were used. To correct the quantitative maps for the effect of RF transmit inhomogeneities, we measured the transmit field B1 + using 3D echo-planar imaging (EPI) spin-echo (SE) and stimulated echo (STE) images. The EPI images were acquired with the 4 mm isotropic resolution, parallel imaging using GRAPPA factor 2 × 2 in PE and partition direction, TESE/TESTE/TM (mixing time)/TR = 37.06/37.06/31.2/500 ms. A B0 map was acquired to correct the RF transmit field maps for geometric distortion and off-resonance effects. The acquisition protocol used a 2D double-echo FLASH sequence with the following parameters (Lutti et al., 2012, Lutti et al., 2010): slice thickness = 2 mm, TR = 1020 ms, TE1/TE2 = 10/12.46 ms, α = 90°, BW = 260 Hz/pixel and flow compensation. The total acquisition time was 24 min (for details on MRI acquisition parameters see Table 1, Supplementary material).

Quantitative MRI maps were calculated from the acquired data using an in-house code running under SPM12 (Wellcome Trust Centre for Neuroimaging, London, UK; http://www.fil.ion.ucl.ac.uk/spm) and Matlab 7.11 (Mathworks, Sherborn, MA, USA). The R2* maps were calculated from the regression of the log signal from the eight PD-weighted echoes. The signals of six equidistant bipolar gradient echoes were averaged to increase the signal-to-noise ratio (SNR), (Helms and Dechent, 2009) before calculation of the R1 and MT maps as described in (Helms et al., 2009, Weiskopf et al., 2013). The quantitative R1 maps, used to calculate the MT images, were corrected for inhomogeneities in the local RF transmit field (Helms et al., 2008a, Helms et al., 2008b).

To test the effects of the new TPMs on tissue classification based on T1w images, we analyzed a second data set (*n* = 33) consisting of Modified Driven Equilibrium Fourier Transform (MDEFT) T1w images. The data set (20 women, age range = 22–85 years, mean age = 37 ± 13 years; 13 men, age range = 18–73, mean age = 47 ± 19 years) was used in a previous study (Helms et al., 2009). The study participants were neither affected by brain disorders nor showed macroscopic brain abnormalities, i.e., major atrophy, or signs of overt vascular pathology, i.e., micro-bleeds and white matter lesions. The 3D MDEFT images were acquired as follows: TR = 7.92 ms, TE = 2.48 ms, TI = 910 ms (symmetrically distributed around the inversion pulse; quot = 50%), flip angle α = 16, fat saturation, bandwidth 19 Hz/pixel, 1 mm^3^ isotropic resolution, acquisition time ~ 13 min (Deichmann et al., 2004) (for details on acquisition parameters see Table 1, Supplementary material). The interaction between the effect of the new TPMs on the MDEFT classification and age was analyzed with R2* maps acquired on the same cohort using the quantitative protocol described above.

### Atlas-based labeling of midbrain structures

The creation of the new TPMs was based on the manual labeling of midbrain structures. Aiming to facilitate the manual labeling, we used spatial information derived from established brain atlases. We used the Harvard–Oxford atlas for caudate and pallidum (Goldstein et al., 2007), the basal ganglia human area template (BGHAT) for putamen (Prodoehl et al., 2008), and Morel's stereotactic atlas for thalamus and subthalamic nucleus (STN) (Morel et al., 1997). The red nucleus (RN) and the substantia nigra (SN) were labeled on the basis of Talairach's atlas (Lancaster et al., 2000), and the cerebellar dentate was labeled according to a spatially unbiased atlas template (SUIT) (Diedrichsen, 2006).

The anatomical labels were non-linearly registered to subject-specific native space using spatial transformation parameters estimated with a diffeomorphic registration algorithm, DARTEL (Ashburner, 2007). To this end, MT saturation maps were processed with the default settings and classified into different tissue classes: gray matter (GM), white matter (WM), cerebro-spinal fluid (CSF), and non-brain tissue, using the “unified segmentation” approach in SPM12 followed by estimation of diffeomorphic registration parameters (Ashburner and Friston, 2005, Ashburner, 2007). This allowed the atlas information to be projected onto each subject's MRI data, prior to the manual labeling procedure that we describe in the following section.

### Manual labeling based on atlas-derived masks

**Fig. 1:**
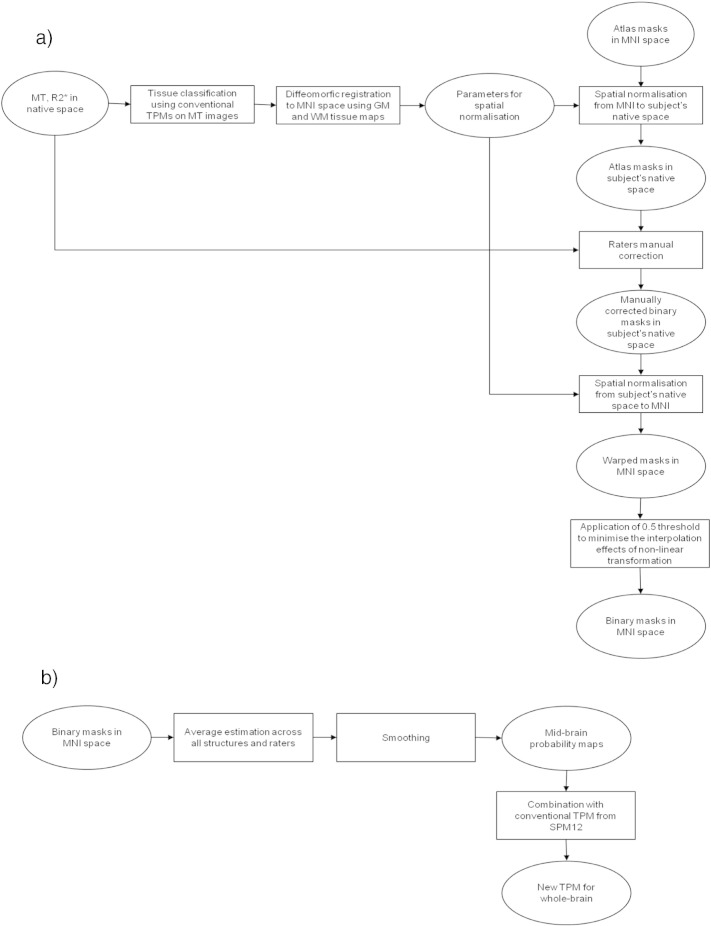
Flowchart summarizing the steps to create the new tissue probability maps (TPM). (a) Atlas information and manual labeling of binary masks relative to subcortical structures. (b) Creation of the new TPM from the binary masks.

The manual labeling was performed using an in-house web-based tool with graphic user interface. The graphic interface allowed visualization of subject-specific MT and R2* images in three principal planes—axial, sagittal, and coronal. The subcortical structure- and hemisphere- specific binary mask of atlas-based labeled voxels was then overlaid on the MT and R2* maps. Four different raters were asked to manually adjust the masks for each subcortical structure according to the subject-specific anatomy. They were instructed to use the MT map to segment the caudate, pallidum, putamen, and thalamus. The extent of STN, RN, cerebellar dentate, and SN was defined from the R2* maps. Manual labeling always started from the axial plane, except for the STN where the initial plane was the coronal (see Fig. 1a for summary of manual labeling procedure).

To assess the inter-rater reliability, we calculated subject- and structure- specific Cohen's kappa, Dice coefficients, and intraclass coefficient (Dice, 1945, Cohen, 1960, Shrout and Fleiss, 1979). Additionally, we estimated the percentage of disagreement between raters, expressed as the ratio between the number of voxel not included by all raters and the number of those labeled at least by one rater.

### Creation of tissue probability maps

To create the TPMs of subcortical regions, the binarized manually labeled masks were spatially registered to standard MNI space using the diffeomorphic spatial transformation parameters estimated for the atlas-labeling step. Aiming to minimize the non-linear effects related to the spatial transformation, we applied a threshold of 0.5 to the masks after the warping step. Then we averaged the masks across all raters. Subsequently, the mean image was smoothed by convolution with an isotropic Gaussian kernel of 4 mm to obtain the midbrain probability (MBP) map. The choice of 4 mm kernel size aimed at preserving borders between neighboring structures while reducing residual registration problems and partial volume effects.

The final step involved the incorporation of the MBP map into the existing set of SPM12 tissue probability maps after voxel-based adjustments, while ensuring a sum of probabilities equal to one across all six tissue priors. Eqs. (1), (2) summarize the aforementioned operations calculated at the single voxel level:

(1) $${\text{newTPM}}_{\mathrm{tc}}={\mathrm{TPM}}_{\mathrm{tc}}\times \left(1-\mathrm{MBP}\right)$$

(2) $${\text{newTPM}}_{\mathrm{gm}}=\mathrm{MBP}+{\text{newTPM}}_{\mathrm{gm}}$$

where MBP stands for midbrain nuclei probability and corresponds to the probability estimated from the manual labeling procedure, tc indicates the tissue class (i.e., GM, WM, CSF, and external brain tissues), TPM stands for the conventional tissue probability map, and new TPM represents the new tissue probability map (see Fig. 1b for description of steps to create new TPMs).

### Validation of the new TPMs on MT data

To assess the anatomical accuracy of the tissue classification achieved with the new TPMs, we performed a leave-one-out cross-validation between the results of the manual labeling and of the automated classification obtained from an MT map, which had been excluded from the creation of the TPMs. The tissue classification was carried out using both new and conventional TPMs within SPM12's “unified segmentation” framework (for details on the parameters of “unified segmentation,” see Table 2, Supplementary material). The same procedure was repeated for all MT maps in the data set.

We measured and statistically compared the volumes of midbrain structures present on the GM volume maps derived from the conventional and new TPMs. For every subject the midbrain structures volumes were calculated only from voxels labeled by all raters showing GM probability equal or bigger than 0.2. We compared the volumes per structure using paired *t*-test.

Next, we estimated the Dice coefficient as a measure of overlap between the manually segmented subcortical structures and the corresponding voxels in the GM maps obtained with both TPMs. Identical to above, we considered only voxels labeled identically by all raters. The Dice coefficient was calculated after applying a threshold of 0.2 and 0.5 on the GM maps. The thresholds were used to minimize potential partial volume effects that might affect the overlap between the automatic tissue classification and the manual labeling. Using a paired *t*-test we compared the Dice coefficients obtained for the GM maps estimated with new and conventional TPMs for each structure. We estimated the effect of age and gender on the differences between the Dice coefficients calculated for the two different TPMs using a general linear model. Significance levels were set at *p* < 0.05 after family-wise error (FWE) correction for multiple comparisons.

### Gray matter volume estimation with new TPMs from MDEFT images

To estimate the effects of our new TPMs on conventional voxel-based morphometry (VBM) analysis, we used an independent T1w data set (*n* = 33). We estimated GM volume maps from MDEFT T1w images using conventional and new TPMs. Beyond the main effects we analyzed the interaction between the estimates of GM volume and age.

The MDEFT images were processed in SPM12 with the identical default settings using both new and conventional TPMs. To maximize the anatomical precision, we calculated spatial transformation parameters using DARTEL on GM and WM tissue maps estimated with the new TPMs (Ashburner, 2007). The warped GM probability maps were then scaled by the Jacobian determinants of deformation fields to account for local compression and expansion due to linear and non-linear transformations to create GM volume maps (Ashburner and Friston, 2000). The GM volume maps were then smoothed by convolution with an isotropic Gaussian kernel of 6 mm full-width-at-half-maximum (FWHM).

For statistical comparison of GM volume differences related to TPM local effects, we included all MDEFT-based GM volume maps in the same flexible-factorial design with regressors for age, gender, and total intracranial volume (TIV). To test for interaction between age and TPMs, GM volumes first detrended the data for the effects of gender and TIV. Subsequently, we performed a voxel-wise paired *t*-test between the age regressors estimated for GM volume maps derived with the new and the conventional TPMs. The whole-brain search volume for statistical analysis included the entire cortex and all subcortical brain structures. Regional differences were examined by creating voxel-wise statistical parametric maps for the entire extent of the search volume using the general linear model (GLM) and random field theory (Friston et al., 1994). Significance levels were set at *p* < 0.05 after family-wise error (FWE) correction for multiple comparisons.

### Regression mode

We tested the hypothesis that the tissue classification results provided by the new TPMs were less sensitive to age-related contrast decrease in MRI images driven by changes in local tissue properties (Lorio et al., 2014). To this end, we carried out a linear regression analysis between GM volume, R2*, and age. We performed a voxel-based regression within regions where the paired *t*-test showed differential age-related GM volume loss between conventional and new TPMs.

First, the R2* maps were spatially registered to standard MNI space using subject-specific diffeomorphic estimates, derived in the previous step for the MDEFT images, without scaling by the Jacobian determinants. A combined probability weighting and Gaussian smoothing procedure (Draganski et al., 2011) was used with a 6 mm FWHM isotropic smoothing kernel.

Then we calculated the GM volume differences between the new and conventional TPMs according to the following equation:

(3) $$\Delta \mathrm{GM}={\mathrm{GM}}_{\text{newTPM}}-{\mathrm{GM}}_{\text{oldTPM}}$$

where GM_newTPM_ and GM_oldTPM_ are the GM volume maps estimated from the new and conventional TPMs.

Finally, we implemented a linear regression to evaluate the correlation between volume differences and R2* values:

(4) $$\Delta \mathrm{GM}=\beta R_{2}^{*}+\varepsilon$$

where *β* is the coefficient weighting the contribution of iron content expressed by R2* values and *ε* represents the residuals of the model.

The model was set to determine the β-parameter and residuals at each voxel. To assess the quality of parameter estimation, we calculated *t*-values, testing against the null hypotheses that β were equal to zero. The statistical significance level was set at *p*_FWE_ < 0.05.

Additionally, we investigated the age-related effects on R2* maps using linear regression within the GLM framework of SPM12.

## Results

### Inter-rater reliability

The mean Cohen's kappa ranged between 0.70 and 0.87 across structures, while the mean Dice coefficient was between 0.65 and 0.87 (see Table 1). The mean intraclass coefficient ranged between 0.64 and 0.87, indicating that the manually segmented structures have good inter-rater agreement (see Table 1). The structures exhibiting lower Cohen's kappa, Dice index, and intraclass coefficient were the ones with the highest percentage of voxel label disagreement across raters (see Table 1).

### Validation of the new TPMs on MT images

**Fig. 2:**
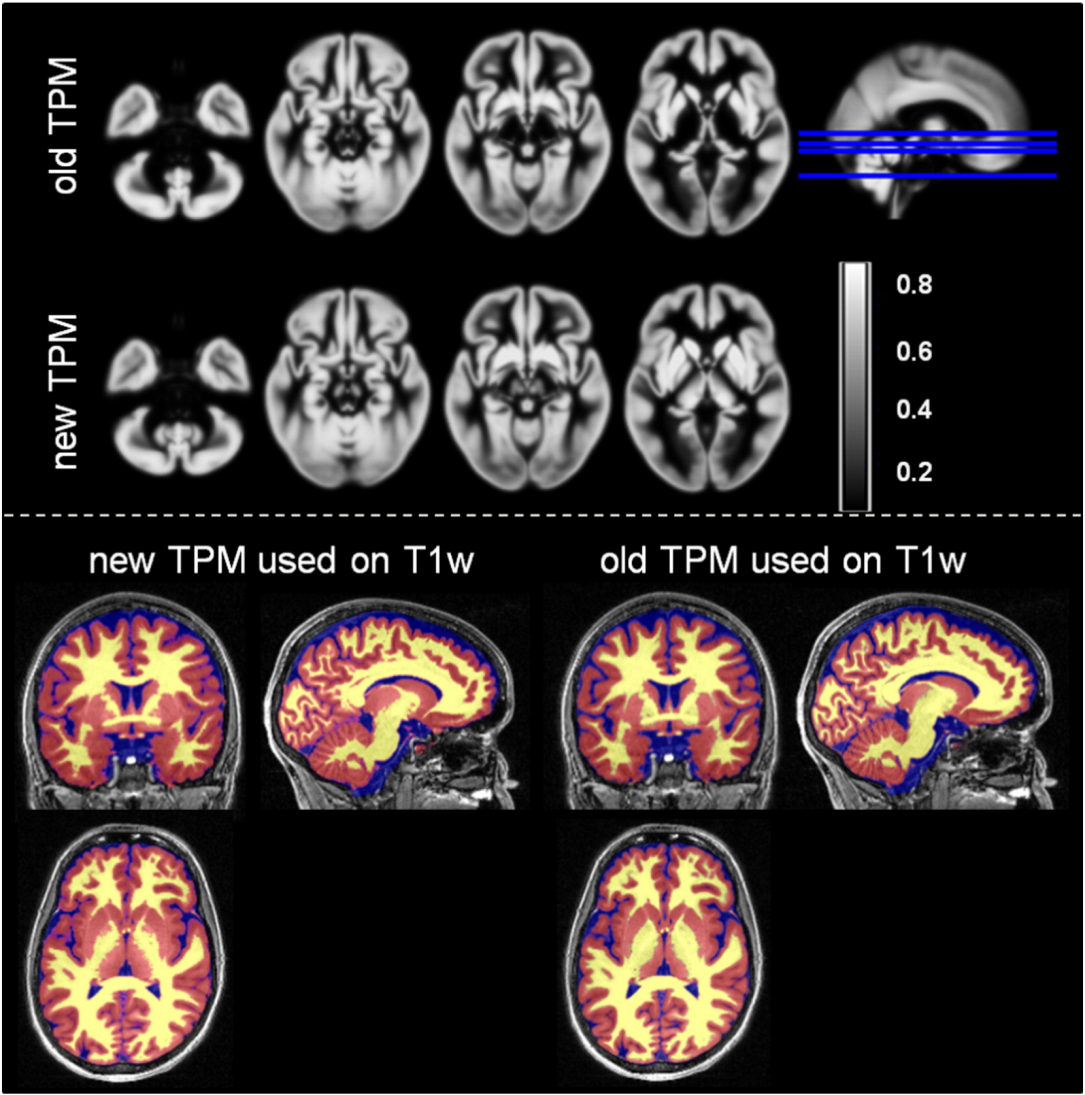
Top panel: axial view on the new and the conventional tissue probability maps (TPMs) for gray matter. Bottom panel: example of tissue classification from T1-weighted data using the new and the conventional TPMs. The gray matter probability maps is represented in red, the white matter—in yellow and the cerebro-spinal fluid (CSF)—in blue.

The usage of the new TPMs (Fig. 2) was associated with greater gray matter volume when compared with estimates based on the conventional TPMs (Table 2). The MT-based GM maps, estimated using the new TPMs, showed a greater overlap with the manually delineated structures with respect to the GM maps calculated with the conventional TPMs (see Table 2).

We found a positive correlation between the Dice coefficients differences and age for caudate, red nucleus, and putamen, bilaterally (see Table 2). The regression analysis showed also a positive correlation between gender and Dice coefficients differences for the caudate and putamen, and a negative correlation for the red nucleus (see Table 2).

### Gray matter volume estimation with new TPMs from MDEFT images

**Fig. 3:**
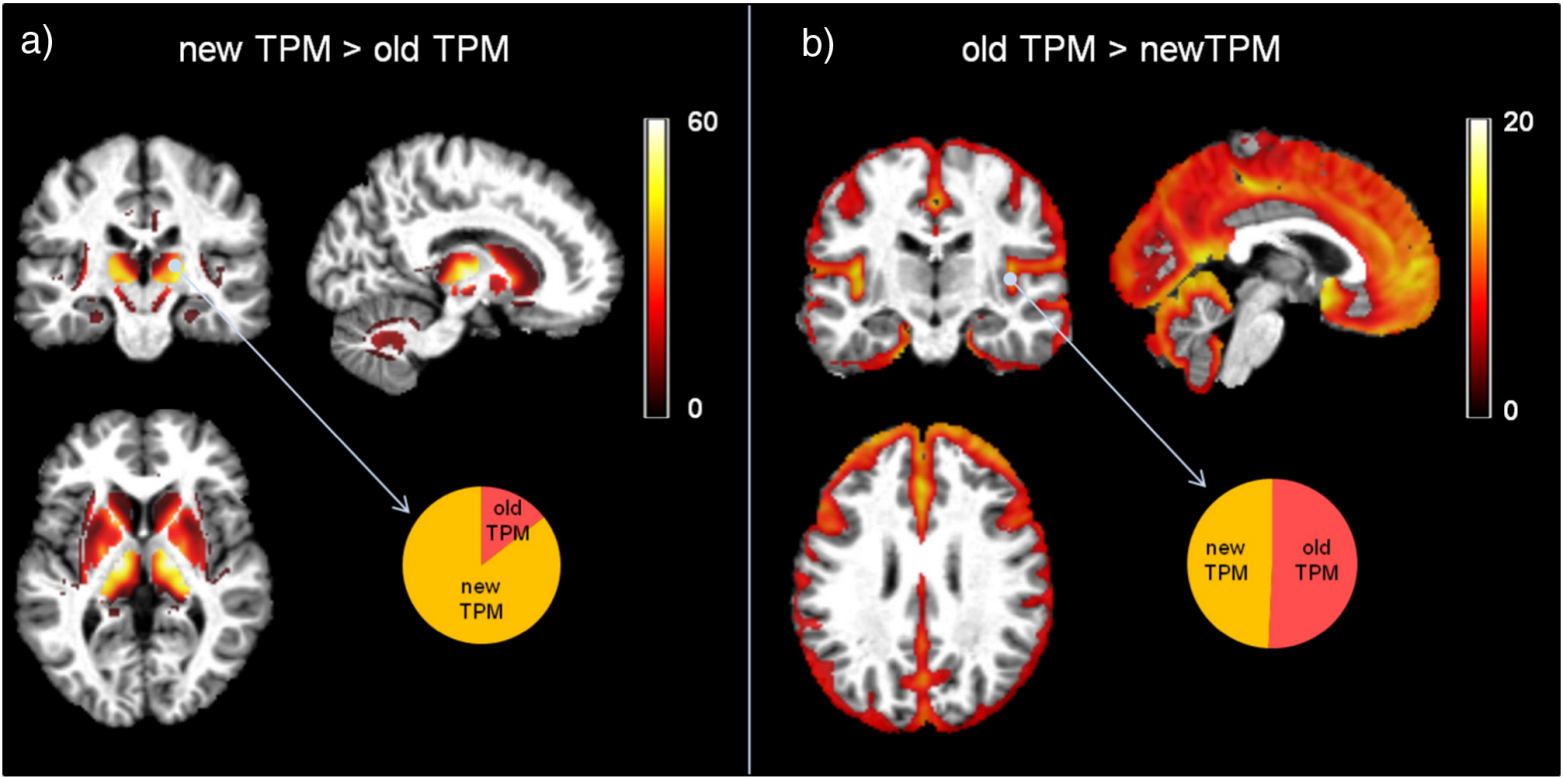
Statistical parametric maps of paired *t*-test between gray matter (GM) volumes estimated from T1-weighted data with new and conventional tissue probability maps (TPM) at statistical threshold of *p*_FWE_ < 0.05. (a) Increase of GM volume estimation, based on the new TPM, compared to the estimation based on conventional TPM. (b) Increase of GM volume estimation, based on the old TPM, compared to the estimation based on conventional TPM. The pie charts represent the effect size for the indicated brain location. All results are presented at *p*_FWE_ < 0.05.

The voxel-based statistical analysis showed higher GM volumes in the striatum, thalamus, and cerebellar dentate with the new TPMs (*p*_FWE_ < 0.05) (see Fig. 3a and Table 3). The use of conventional TPMs resulted in higher GM volume estimation in superficial cortical layers (*p*_FWE_ < 0.05) (see Fig. 3b). We note that the effect size differences between the conventional and new TPMs were comparatively lower in cortical than areas subcortical areas (see Fig. 3).

### Effects of age

**Fig. 4:**
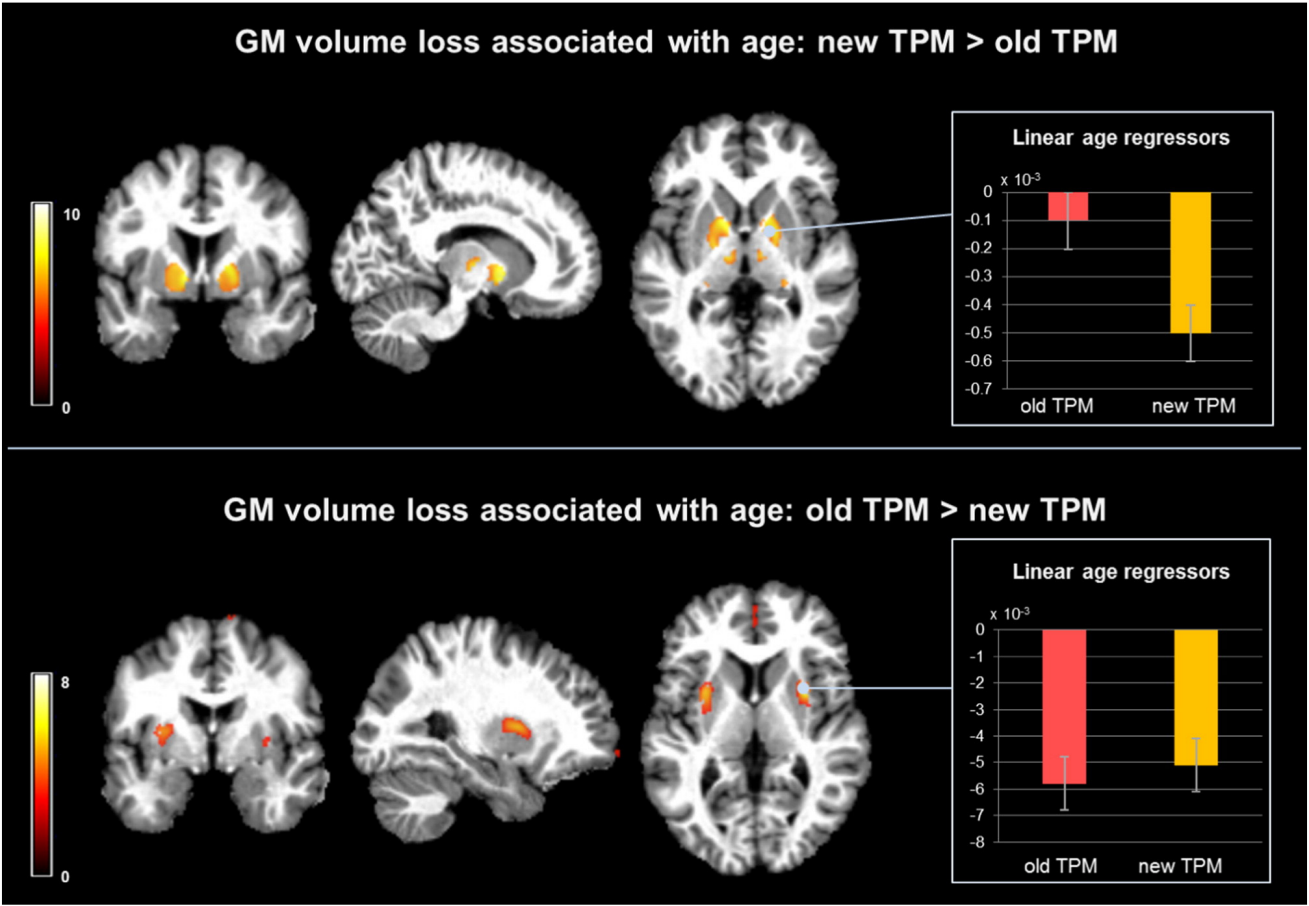
Interaction between age-related gray matter (GM) volume loss and tissue probability maps (TPM) used for GM volume estimation from T1-weighted data. Top panel: statistical parametric maps of stronger negative correlation between age and GM volume estimates from new TPM compared to old TPM. Bar plot—mean linear regressors for age effects on volume in the pallidum. Bottom panel: statistical parametric maps of stronger negative correlation between age and GM volume estimates from old TPM compared to the new TPM. Bar plot—mean linear regressors for age effects on volume in the putamen.

We found a significant (*p*_FWE_ < 0.05) widespread pattern of age-associated GM volume decrease in putamen, caudate, and frontal cortical regions using the new and conventional TPMs on the MDEFT images. There was a greater age-related volume loss in the ventral pallidum when analysing GM maps estimated with the new TPMs and in the dorso-lateral putamen when using the conventional TPMs (see Fig. 4, Fig. 1a, Supplementary material).

**Fig. 5:**
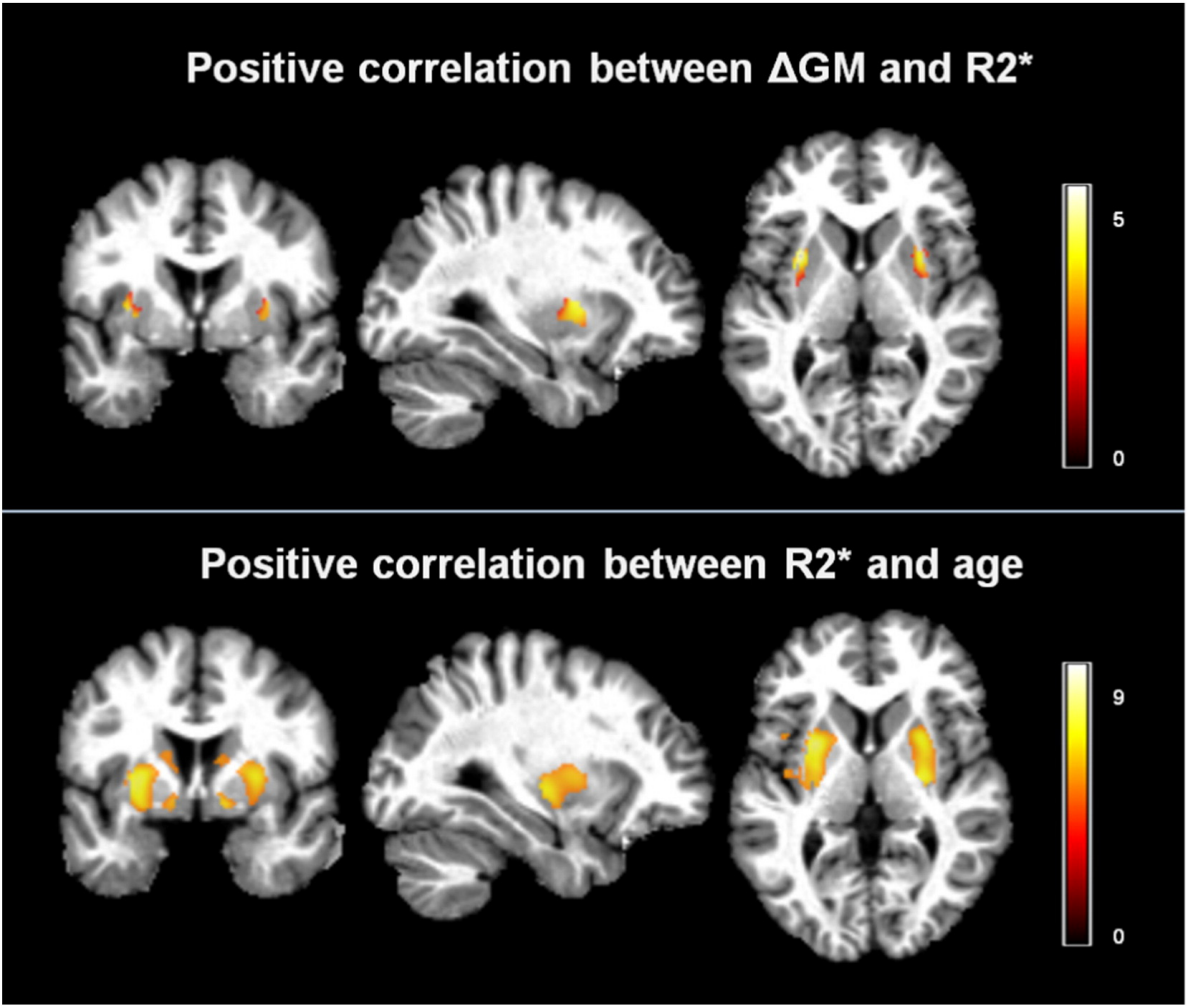
Top panel: *t*-values of voxel-based regressors correlating R2* with higher age-related gray matter (GM) volume loss estimated with conventional tissue probability maps (TPM). Bottom panel: *t*-values for the positive linear correlation between R2* and age in GM voxels.

We report a significant (*p*_FWE_ < 0.05) positive linear correlation between the age-related GM volume differences and the R2* maps in the voxels where conventional TPMs resulted in greater GM volume loss (see Fig. 5). The R2* values of these voxels were positively correlated with age (*p*_FWE_ < 0.05) (see Fig. 5, Fig. 1b, Supplementary material).

## Discussion

Here we create new tissue probability maps (TPMs) of subcortical structures leading to improved anatomical plausibility of automated brain tissue classification when using T1w images. The new TPMs were obtained after manual labeling of subcortical structures from MT and R2* maps, respectively, biomarkers of myelin and iron content, exhibiting optimal tissue contrast for the deep brain nuclei. We emphasize that while the new TPMs were created using two different MRI contrasts, the primary purpose of the new TPMs is the tissue classification of unimodal structural MRI data. Our new TPMs accurately classify the basal ganglia and thalamus in MRI data with different gray-white matter contrast—MT maps and MDEFT T1w images, illustrating the versatility of the new TPMs. The novelty of our study goes beyond the reliable automated tissue classification of previously undetectable subcortical structures. We demonstrate the robustness of the new priors against age-related brain tissue property changes—a limitation of current TPM that has led to the detection of spurious gray matter volume changes in computational anatomy studies.

The consistency of the manual labeling across different raters was in line with previous studies (Ahsan et al., 2007, Babalola et al., 2009, Keuken et al., 2014). Small nuclei such as the subthalamic and red nucleus showed decreased inter-rater consistency due to the lower detectability of these regions on eye inspection and to their close proximity to other nuclei (Keuken et al., 2013). The high spatial overlap between the manual labeling and the automated tissue classification using our new TPMs supports the feasibility of reliable automated classification of structures that were not detectable up to date. Nevertheless, the higher percentage of disagreement for small structures could potentially overestimate the overlap between automated tissue classification and manual labeling due to the fact that less voxel were used for comparison.

Given the widespread use of T1w MRI data for computational anatomy studies (Good et al., 2001, Ashburner et al., 2003), we tested whether the new TPMs achieved better accuracy than current classification on such MRI data. We demonstrate improved anatomical plausibility, particularly for the red nucleus, subthalamic nucleus, and cerebellar dentate, which are not included in old TPMs. Similarly, significant parts of the pallidum, putamen, thalamus, and substantia nigra were accurately detected as GM structures. We note that the classification of these areas as GM by the new TPMs promotes the inclusion of voxel with high signal intensities in the GM intensity distributions over the whole-brain T1w image. This effect may represent the principal cause for the estimation of bigger insula and hippocampus GM volumes obtained with the new TPMs. The shift of GM intensity distributions toward higher values is also expected to skew the classification of low intensity voxel as CSF that may underlie the observed reduction in GM volume in the outer cortical ribbon. It is of note that the effect size differences between old and new TPMs for the cortical contrast was small, which questions any significant impact of the new TPMs on differential estimation of cortical GM volume.

In a proof-of-concept VBM analysis of T1w data, we demonstrate the robustness of the new TPMs against spurious gray matter volume differences due to age-related microstructural tissue changes. Previous studies have demonstrated the profound effect of age on brain anatomy with a robust pattern of cortical changes next to controversial results for subcortical areas. GM estimates derived from the new TPMs show age-related volume loss in cortical regions consistent with previous findings (Good et al., 2001, Fjell et al., 2009, Draganski et al., 2011, Callaghan et al., 2014, Storsve et al., 2014). However, we find relatively preserved GM volume in the dorso-lateral putamen with increasing age compared to previous reports based on old TPMs that highlighted this subcortical region as being the most affected by healthy ageing (Cherubini et al., 2009, Draganski et al., 2011, Callaghan et al., 2014, Oh et al., 2014). The regression model presented here showed that this discrepancy can be largely explained by an age-related increase in iron concentration, as described by the MRI parameter R2*, emphasizing the impact of microstructural changes on the detection of spurious apparent GM volume change. Conversely, we interpret the greater age-related volume loss in the ventral pallidum when using volume estimates based on the new TPMs as result due to the improved classification of pallidum as a gray matter structure. The automated detection of the pallidum—one of the iron-richest structures in the brain (Hallgren and Sourander, 1958), is affected by age-related loss of gray-white matter contrast in T1w images. Correspondingly, the T1w- based current tissue priors for gray matter in the framework of SPM do not include the pallidum, which results in its classification as white matter structure. This interpretation is supported by our findings that highlight the improved classification of pallidum both as a main effect of the TPMs (Fig. 3) and interaction between TPM and age (Fig. 4—top panel).

Age is associated with a linear increase of iron in the subcortical structures, which is confirmed by histopathology and imaging studies directly measuring iron content or estimating it indirectly with the effective transverse relaxation rate R2* (Hallgren and Sourander, 1958, Aquino et al., 2009, Langkammer et al., 2010, Daugherty and Raz, 2015). Iron decreases the gray-white matter contrast of T1w images (Helms et al., 2009, Raz et al., 2005, Lorio et al., 2014) and impacts the automated classification of brain tissue types, which heavily relies on between-tissue intensity differences. While this increase in iron concentration with age was observed over the entire putamen, age-related volume differences across TPMs could only be detected in its dorso-lateral part. This is consistent with differences in MR contrast, which are most prominent at the interface between neighboring tissues. This regional specificity suggests that other tissue characteristics such as fiber loss and axonal damage might play a role in the volume reduction of that region (Cherubini et al., 2009).

Given all these considerations, we conclude that the GM volume maps estimated with the new TPMs from T1w images are less sensitive to age-related gray-white matter contrast changes and are more suitable for accurate representation of the dynamics of age-associated brain anatomy changes. Our new TPMs are derived from a fairly large cohort of subjects with a broad age range. This assures the inclusion of many brain changes occurring with increasing age, such as iron accumulation in subcortical regions (Hallgren and Sourander, 1958) and increase of ventricular size (Fjell and Walhovd, 2010).

### Limitations and outlook

The new TPMs were created and tested solely on data from healthy subjects, which limits the main and interaction effects to the case of normal ageing across gender. Any tissue property change, particularly abnormal iron deposition which is a hallmark of neurodegeneration, will have a more profound and differential spatially distributed effects on brain structure.

We also acknowledge that region delineation based on *postmortem* myelo- and cyto-architectonic assessment provides greater specificity and accuracy in tissue border definition (Deistung et al., 2013) than the approach chosen here. In this study, the finite resolution of the image voxels leads to partial volume effects and blurring of the borders between neighboring structures, limiting the accuracy of the manual labeling and of automated tissue classification. However, in the absence of a ground truth provided by individual histological maps, the quantification of such effects is very difficult.

Our new TPMs showed a clear improvement in the automated classification of subcortical structures from MRI data with optimal contrast in these regions and from broadly used T1-weighted data. The newly created TPM is readily usable in the established framework of the SPM software. We have empirically assessed the robustness of the new TPMs against the effects of age-related microstructural tissue changes on tissue classification, preventing the detection of spurious apparent volume change in neuroanatomy studies. The new TPMs can be used for studying effects on the healthy brain by disease, particularly when the emphasis is on subcortical structures. Future work linking automated tissue classification and underlying histological properties will help validate and extend the generalizability of this study.

## Figures and Tables

**Table 1:** Manual labeling results. Subcortical structures' mean volume, global percentage of voxels not included by all raters (disagreement voxels), and inter-rater agreement indices (Dice index, Cohen's kappa, and intraclass coefficient (ICC)). RN = red nucleus; STN = subthalamic nucleus; SN = substantia nigra; GP = globus pallidus.

Structure		Volume (mm^3^)		% of disagreement voxels		Dice index		Cohen's kappa		ICC	
Mean	SD	Mean	SD	Mean	SD	Mean	SD	Mean	SD
Caudate	Left	3421	900	17	3	0.83	0.06	0.85	0.06	0.83	0.06
Right	3306	700	16	3	0.85	0.06	0.86	0.06	0.85	0.06
Putamen	Left	3906	650	19	3	0.80	0.05	0.8	0.05	0.83	0.05
Right	3966	690	18	4	0.85	0.03	0.86	0.03	0.84	0.04
GP	Left	1319	235	20	4	0.79	0.08	0.8	0.08	0.78	0.08
Right	1263	201	21	5	0.76	0.09	0.77	0.09	0.77	0.09
Thalamus	Left	5110	1100	16	4	0.86	0.04	0.86	0.04	0.86	0.04
Right	5495	1301	15	3	0.87	0.05	0.87	0.05	0.87	0.04
SN	Left	330	94	25	6	0.7	0.11	0.74	0.12	0.67	0.11
Right	330	90	23	5	0.76	0.14	0.77	0.14	0.68	0.12
RN	Left	220	49	29	7	0.68	0.13	0.71	0.13	0.64	0.1
Right	220	50	28	8	0.69	0.11	0.77	0.11	0.67	0.11
STN	Left	86	28	33	7	0.65	0.14	0.70	0.12	0.67	0.1
Right	85	20	31	7	0.7	0.1	0.73	0.19	0.69	0.1
Dentate	Left	1032	215	20	5	0.76	0.11	0.73	0.11	0.7	0.11
Right	1013	195	23	6	0.77	0.14	0.76	0.13	0.69	0.12

**Table 2:** Validation of the new tissue probability maps (TPMs) on MT images. Gray matter volume of subcortical structures estimated with old and new TPMs. *t*-value Vol_newTPM_ > Vol_oldTPM_—corresponding *t*-values for comparison between volumes estimated with new and old TPM. Dice index (DI) of overlap between manual labeling and automated segmentation at voxel-based threshold of 0.2 (Th 0.2) and 0.5 (Th 0.5). *t*-value DI_newTPM_ > DI_oldTPM_—corresponding *t*-values for comparison between Dice index estimated for the automated segmentation with new and old TPM. *t*-value ΔDI vs age/sex—corresponding *t*-values for the linear correlation of the Dice index differences with age and sex. We report only *t*-values with *p*_FWE_ < 0.05. Th—absolute threshold of gray matter maps at 0.2 resp. 0.5. RN = red nucleus; STN = subthalamic nucleus; SN = substantia nigra; GP = globus pallidus.

Structure		Volume (mm^3^) old TPM (mean ± SD)	Volume (mm^3^) new TPM (mean ± SD)	*t*-value Vol_newTPM_ > Vol_oldTPM_	Dice index old TPM Th 0.2 (mean ± SD)	Dice index new TPM Th 0.2 (mean ± SD)	Dice index old TPM Th 0.5 (mean ± SD)	Dice index new TPM Th 0.5 (mean ± SD)	*t*-value DI_newTPM_ > DI_oldTPM_ Th 0.2	*t*-value DI_newTPM_ > DI_oldTPM_ Th 0.5	*t*-value ΔDI vs age Th 0.2	*t*-value ΔDI vs sex Th 0.2	*t*-value ΔDI vs age Th 0.5	*t*-value ΔDI vs sex Th 0.5
Caudate	Left	3181 ± 396	4	3482 ± 310	0.92 ± 0.02	0.99 ± 0.01	0.95 ± 0.10	0.97 ± 0.10	10.8	13.0	6.8	5.9	4.1	3.6
Right	2838 ± 399	5	3420 ± 305	0.93 ± 0.01	0.99 ± 0.01	0.95 ± 0.10	0.98 ± 0.10	10.4	4.9	7	6.2	4.37	3.87
Putamen	Left	2879 ± 470	4.8	3372 ± 401	0.90 ± 0.01	0.99 ± 0.01	0.94 ± 0.06	0.97 ± 0.04	14.1	10.0	5	7.3	5.4	4.1
Right	2870 ± 470	4.3	3598 ± 401	0.90 ± 0.01	0.99 ± 0.01	0.94 ± 0.06	0.97 ± 0.04	14.5	9.4	6	6.3	5.82	4.86
GP	Left	402 ± 112	17.8	1032 ± 203	0.30 ± 0.15	0.75 ± 0.15	0.25 ± 0.15	0.63 ± 0.16	36.4	32.6	–	–	–	–
Right	373 ± 130	18.2	1195 ± 211	0.30 ± 0.17	0.75 ± 0.14	0.25 ± 0.15	0.63 ± 0.16	45.4	35.7	–	–	–	–
Thalamus	Left	3160 ± 473	14.1	4876 ± 705	0.6 ± 0.15	0.86 ± 0.06	0.53 ± 0.08	0.80 ± 0.07	51.8	49	–	–	–	–
Right	3791 ± 547	14.3	5077 ± 837	0.56 ± 0.1	0.82 ± 0.06	0.50 ± 0.09	0.78 ± 0.07	39.8	48.7	–	–	–	–
SN	Left	226 ± 45	10	293 ± 52	0.48 ± 0.14	0.78 ± 0.13	0.30 ± 0.14	0.68 ± 0.14	38.4	38.4	–	–	–	–
Right	242 ± 61	9.5	316 ± 66	0.44 ± 0.16	0.77 ± 0.13	0.31 ± 0.15	0.69 ± 0.16	32.9	32.3	–	–	–	–
RN	Left	7 ± 4	12.7	50 ± 10	0.02 ± 0.01	0.32 ± 0.09	0.01 ± 0.01	0.22 ± 0.01	14.8	10.2	11.13	− 7.8	5	− 5.39
Right	6 ± 4	13.8	52 ± 10	0.02 ± 0.01	0.34 ± 0.09	0.01 ± 0.01	0.23 ± 0.01	14.3	10.2	11.7	− 6.8	5.3	− 4.53
STN	Left	9 ± 6	8.5	32 ± 7	0.04 ± 0.01	0.25 ± 0.01	0.02 ± 0.01	0.22 ± 0.01	10.7	9.6	–	–	–	–
Right	9 ± 4	10.7	33 ± 8	0.04 ± 0.01	0.25 ± 0.01	0.03 ± 0.01	0.22 ± 0.01	12.3	10.6	–	–	–	–
Dentate	Left	122 ± 60	16.8	977 ± 140	0.25 ± 0.01	0.65 ± 0.11	0.16 ± 0.01	0.51 ± 0.11	26.5	21.8	–	–	–	–
Right	106 ± 50	15.5	987 ± 125	0.27 ± 0.01	0.69 ± 0.13	0.18 ± 0.01	0.56 ± 0.13	20.5	14	–	–	–	–

**Table 3:** Statistical comparison of gray matter volume estimates. Voxel-based comparison between gray matter volume maps estimated with new and conventional tissue probability maps (TPM) using T1-weighted MDEFT images. Coordinates are given in Montreal Neurological Institute (MNI) standard space. SN = substantia nigra; GP = globus pallidum.

	Structure	Left hemisphere coordinates (mm)			*t*-value	Cluster size (number of voxels)	Right hemisphere coordinates (mm)			*t*-value	Cluster size (number of voxels)
*x*	*y*	*z*			*x*	*y*	*z*		
New TPM > Old TPM	GP	− 15	9	3	47.39	6195	16	− 4	− 1	30	6483
Caudate	− 12	13	6	47.39	12	13	6	44
Putamen	− 27	0	12	45.53	28	0	12	44.39
Thalamus	− 14	− 15	3	74.71	2411	18	− 15	6	74.22	2443
SN	− 4.5	− 12	− 10.5	19.54	120	6	− 12	− 9	18.01	134
Dentate	− 14	− 51	− 35	18.01	1357	17	− 63	− 32	19.68	1294
