# Supplementary material for: New tissue priors for improved automated classification of subcortical brain structures on MRI
Source: Neuroimage. 2016 Apr 15;130:157–66. doi: 10.1016/j.neuroimage.2016.01.062 (PMC4819722; doi:10.1016/j.neuroimage.2016.01.062)
Supplement: Supplementary file 1 — Supplementary material. [file mmc1.docx]

**Title:** New tissue priors for improved automated classification of subcortical brain structures on MRI

**Supplementary material**

| **Sequence** | **TR (ms)** | **TE (ms)** | **TI (ms)** | **α (deg)** | **Resolution (mm)** | **Field of view (mm)** | **Acquisition time (min)** |
| --- | --- | --- | --- | --- | --- | --- | --- |
| **MPM 3D FLASH T1-weighted** | 18.7 | 6 equidistant between 2.2 and 14.7 | -- | 20° | 1 isotropic | 256×240×176 | 6 |
| **MPM 3D FLASH MT-weighted** | 23.7 | 6 between 2.2 and 14.7 | -- | 6° | 1 isotropic | 256×240x176 | 6 |
| **MPM 3D FLASH PD-weighted** | 23.7 | 8 equidistant between 2.2 and 19.7 | -- | 6° | 1 isotropic | 256×240×176 | 7 |
| **RF transmit field map: 3D EPI SE and STE** | 500 | TE_SE_/TE_STE_ = 37.06/37.06 | -- | α_SE/STE_ decreased from 230°/115° to 130°/65° in steps of 10°/5° | 4 isotropic | 256×192×192 | 3 |
| **B0 map: 2D FLASH** | 1020 | 10/12.46 | -- | 90° | 3x3x2 | 192x192 | 2 |
| **MDEFT T1-weighted** | 7.92 | 2.48 | 910 | 16° | 1 isotropic | 256×240×176 | 13 |

Table 1: Acquisition parameters of the multi-parametric mapping (MPM), radiofrequency (RF) transmit field, static magnetic field (B0) mapping and T1-weighted MDEFT images. TR = repetition time, TE = echo time, TI = inversion time, α = flip angle, SE = spin echoes, STE = stimulated echoes.

| **Tissue classification parameters** | | **Number of Gaussian** | **Bias regularisation** | **Bias FWHM** | **MRF parameter** |
| --- | --- | --- | --- | --- | --- |
| **Tissue class** | **Grey matter** | 2 | -- | -- | -- |
|  | **White matter** | 1 | -- | -- | -- |
|  | **CSF** | 2 | -- | -- | -- |
|  | **Bone** | 3 | -- | -- | -- |
|  | **Soft tissue** | 4 | -- | -- | -- |
|  | **Background** | 2 | -- | -- | -- |
| **Intensity Bias** | | -- | 0.001 | 60mm cut-off | -- |
| **MRF** | |  |  |  | 1 |

Table 2: “Unified segmentation” parameters used for the tissue classification of MT and T1-wieghted images with old and new TPM.

**
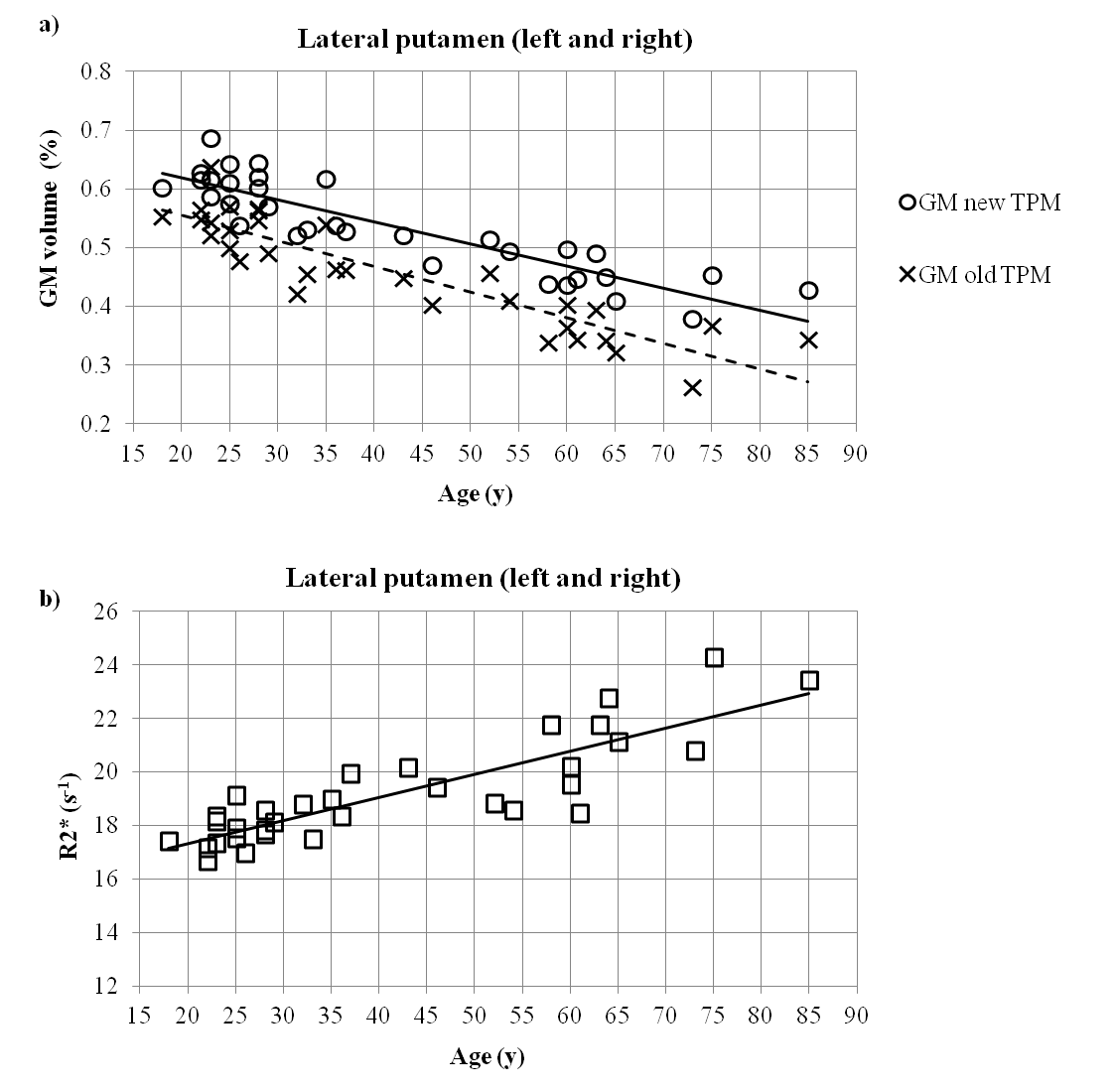
**

Figure 1: Scatter plots of age-related grey matter (GM) volume and R2* differences. a) Mean GM volume estimated applying the new TPM (circles) and conventional TPM (crosses) on MDEFT T1w data. Mean GM values extracted from the dorso-lateral putamen (bottom panel Fig 4). Linear regression between age and GM volumes derived with new TPM (solid line), linear regression between age and GM volumes derived with old TPM (dashed line). b) Age-related increase of R2*; mean R2* values extracted from the dorso-lateral putamen (bottom panel Fig. 4).
